# Grand Challenges in AI in Radiology

**DOI:** 10.3389/fradi.2021.629992

**Published:** 2021-04-13

**Authors:** Tianming Liu

**Affiliations:** Department of Computer Science, University of Georgia, Athens, GA, United States

**Keywords:** artificial intelligence, imaging, radiology, deep learning, data sharing

AI technologies and methodologies have started to empower all aspects of radiology in the past decade, from imaging data acquisition, to imaging data interpretation, and to clinical decision making. Despite exciting advancements made in the field of AI in radiology, there are also grand challenges associated with technological and translational aspects of AI in radiology (1). Following the Field Chief Editor Dr. Dinggang Shen's perspective in his statement of grand challenges in radiology^1^, here we elaborate four grand challenges in AI in radiology in more detail.

## Grand Challenges in AI-Empowered Imaging Data Acquisition

Fast and high-quality radiological image acquisition has been a major challenge for decades and it remains as a grand challenge. How to speed up imaging data acquisition, such as MRI and CT scans, has been of major interest to improve efficiency and patient safety, among other considerations. In response, many AI technologies have been developed and reported for fast and high-quality radiological image reconstruction (2), with substantially smaller doses of intravenous contrast material and lower radiation dose in some scenarios. It is envisioned that these new imaging data acquisition technologies will continue to be developed for the benefits of patients, radiologists, and radiology clinical flow. Also, AI can play a major role in integrating and optimizing radiological data acquisition workflow, for instance, a recent successful example is the contactless patient positioning system during the COVID-19 pandemic (3), which automated calibration, positioning, and multi-view synthesis components that enable patient scans without physical proximity. This journal's specialty of AI in radiology will encourage and welcome contributions that address all aspects of AI-empowered imaging data acquisition.

## Grand Challenges in Data Annotation for AI Algorithm Training

Modern AI systems in radiology typically rely on machine learning and deep learning algorithms that are trained and tested on a large number of annotated radiological images (4). Thanks to significant progresses made in the AI field, algorithms can now take advantage of accurately and reliably annotated imaging data. However, manual annotation of radiological images is still a key bottleneck in translating advanced AI algorithms into clinically useful systems. Typically, manual labeling and annotation processes in radiology AI systems are quite costly and time-consuming. Thus, developing effective automated labeling and annotation methods to produce high-quality training and testing data for radiology AI research and application is much needed (1). Recent efforts in integrating natural language processing (NLP) technologies and human-computer interaction (HCI) methodologies into radiology will be a promising direction to explore and pursue in the future.

## Grand Challenges in Radiology Data Sharing

Although radiology AI systems are deemed to be promising in improving efficiency and accuracy, they require a sufficiently large, well-curated, well-integrated, and controlled dataset for training and testing AI algorithms. In particular, radiology AI systems for many human diseases/conditions are heavily dependent on a variety of co-factors such as disease stages or subtypes, patient populations, and genotypes, among many others. Given the large number of combinations of the co-factors, the data from one single institution are vastly insufficient for AI algorithms to achieve their full potential. In addition, in order to have complete and diversified data to minimize healthcare disparities, radiological data sharing among multiple institutions is required to meet such requirements. Unfortunately, the concerns of data security and patient privacy in data sharing and other social/economic considerations prevent people from sharing radiological data in a large scale effectively, which significantly prevents the clinical applications of radiology AI systems. This is a grand challenge for radiology AI that truly deserves attention and effort from all stakeholders to overcome. From a technical perspective, recent decentralized approaches such as blockchain and the InterPlanetary File System (IPFS)^2^ possess great promise in dealing with security and privacy concerns in data sharing and thus they are worthy of future exploration for radiological image sharing.

## Grand Challenges in Human Factors in AI in Radiology

Radiology AI systems are used by clinical radiologists during their daily practice, and thus AI systems and radiologists must adapt to each other and build up a co-worker relationship. However, it is known that such human-machine co-working is challenging, which is formulated as the human-technology frontier in the NSF's 10 big ideas^3^. There will be many research opportunities to understand and build the radiologist-AI system relationship, to design and develop new technologies to augment radiologist's performance, and to foster radiologist's lifelong and pervasive learning with AI systems. It is hopeful that these effective AI-radiologists co-working models will significantly increase radiologists' efficiency and reduce AI system errors and risks.

## Author Contributions

TL wrote this article as a sole author.

## Conflict of Interest

The author declares that the research was conducted in the absence of any commercial or financial relationships that could be construed as a potential conflict of interest.

## Footnotes

^1^ https://www.frontiersin.org/journals/radiology#about

^2^ https://ipfs.io/

^3^ https://www.nsf.gov/news/special_reports/big_ideas/
